# Mucormycosis, one month after recovery from COVID-19: A case report

**DOI:** 10.1016/j.amsu.2022.103911

**Published:** 2022-06-04

**Authors:** Shahriar Alian, Fatemeh Ahangarkani, Seyyed Javad Boskabadi, Saeed Kargar-Soleimanabad, Leila Delavarian, Azalia Pakzad

**Affiliations:** a Antimicrobial Resistance Research Center, Communicable Diseases Institute, Mazandaran University of Medical Sciences, Sari, Iran; b Department of Clinical Pharmacy, Faculty of Pharmacy, Mazandaran University of Medical Sciences, Sari, Iran; c Student Research Committee, Faculty of Medicine, Mazandaran University of Medical Sciences, Sari, Iran

**Keywords:** Mucormycosis, Black fungus, Covid-19, Diabetes, Strongyloidiasis

## Abstract

**Introduction:**

and importance: There are increasing case reports of mucormycosis in patient with coronavirus disease 2019 (Covid-19). Herein, we describe the case of mucormycosis after recovery from Covid-19.

**Case presentation:**

The patient was a 73 years old woman with a history of chronic kidney disease, diabetes mellitus, hypertension, and dyslipidemia that referred to the emergency department with clinical presentation of Covid-19. On the third day of admission, the Covid-19 PCR test was negative, but the patient presented headache and pain in her upper jaw. Physical examination showed fever, erythema, and tenderness in the right cheek. Emergency biopsy and culture from sinus by subsection to mucormycosis conducted. and the diagnosis of mucormycosis was confirmed by the positive result of biopsy and culture. Despite anti-fungal treatment with Amphotericin B, patient developed severe diarrhea and became hemodynamically unstable. In the stool analysis, Strongyloides stercoralis was reported. Unfortunately, patient was expired on day thirty-two of this admission.

**Clinical discussion:**

Mucormycosis is a dangerous infection, and its rapid diagnosis is so important. On the other hand, Covid-19 may associated with many nonspecific sign and symptoms. These finding may overlap with other infections.

In patients with prolonged mucormycosis infection, the development of strongyloidiasis should not be neglected. A single dose of ivermectin as strongyloidiasis prophylaxis should be given if the duration of the illness is prolonged.

**Conclusion:**

Clinicians should consider mucormycosis and its complications after Covid-19 treatment in diabetic and immunocompromised patients.

## Highlights

• COVID-19 may be associated with bacterial or fungal co-infections.
• Nonspecific sign and symptoms of COVID-19, may overlap with other infections.
• Mucormycosis may occur following COVID-19, especially in patients with risk factors.
• We report a case of mucormycosis, one month after recovery from COVID-19 in a patient with poor diabetic control.

## 1 Introduction

In December 2019, the new coronavirus was recognized in China's Hubei province. The Coronavirus Disease 2019 (Covid-19) caused Pneumonia and lung infection and expanded quickly in all of the world [1]. In The February 2020, World Health Organization (WHO) Announced infectious coronavirus disease 2019 officially. Until the end of December 2021, more than 290 Million infection to Covid-19 and five million death reported in the world [2,3].

Every individual may exposure to sever infection of coronavirus but the risk is the highest in people older than 60 years old with chronic disease. Hospitalization and mortality rate in patients with chronic disease increased 6 times and 12 times than healthy people respectively [4].

This disease manifested with a very wide range of presentations from asymptomatic disease to pneumonia, even acute respiratory distress syndrome (ARDS) with high mortality rate [5]. Covid-19 may be associated with co-infections, such as the variety of bacterial and fungal infections. It may exist formerly or can develop as new-onset infections. Patients with coronary artery disease, hematological malignancy, diabetes mellitus, organ transplantation and immunocompromised patients are more susceptible to progress to co-infections [6].

Mucormycosis is an opportunistic aggressive invasive fungal infection from the order Mucorales and the zygomycete family. Rhizopus oryzae is the most current pathogen isolated from patients and it's responsible for more than 70% of mucormycosis [7].

Mucormycosis is an uncommon but a fatal angio-invasion fungal infection that may be associated with high mortality rate [8]. Clinical manifestations of mucormycosis are too different and consist of rhino-cerebral, rhino-orbital, pulmonary, gastrointestinal, disseminate, and some other uncommon characteristics [9].

Some underlying disease including uncontrolled diabetes mellitus, iron overload, treatment with deferoxamine, hematologic malignancies, malnutrition, renal and liver disease can predispose to the mucormycosis infection [10,11].

In addition to the above conditions, other underlying diseases may also play a role in the development of this infection. There have been case reports of mucormycosis in patients diagnosed with Covid-19. The majority of these reports suggest that onset of mucormycosis symptoms usually occurs one or two weeks after admission for Covid-19 [12]. However, even long after recovery from Covid-19, the mucormycosis infection may be occur.

This case report discussed mucormycosis following recovery from Covid-19 in a patient with cardiovascular, renal and metabolic risk factors.

## 2 Case presentation

This study was conducted according to the Declaration of Helsinki principles, as well as CARE guidelines and methodology [13].The patient was a 73 years old woman who referred to the emergency department with dyspnea, nausea, vomiting, myalgia weakness and lethargy. Her symptoms started 4 days ago. Swelling and redness of the face were observed on physical examination and she denied any chest pain or palpitation. One month before this presentation, the patient had been diagnosed and admitted by covid-19 for five days.

Her past medical history was chronic kidney disease, diabetes mellitus, hypertension and dyslipidemia. The patient's regular medications included aspirin (80 mg once daily), prednisolone (5 mg once daily), metoprolol (50 mg twice daily), amlodipine, (4 mg twice daily), pantoprazole (40 mg once daily), furosemide (40 mg once daily), losartan (25 mg twice daily), Insulin (60 unites twice daily), calcitriol (0.25 mg daily), nitrocontine (6.4 mg twice daily), clonazepam (1 mg before sleep), salbutamol spray (2 puff, three times a day).

At emergency department, blood pressure was 100/60 mm Hg, heart rate was 70 beats/min, and axillary temperature was 36.5_C and oxygen saturations were 89%. Table .1 demonstrated the initial laboratory data in emergency department. In the lung computed tomography (CT) -scan, suspected lesions was observed. However, it was not possible to distinguish whether these lesions were new or related to previous Covid-19 infection. Because of the clinical symptoms and pandemic Covid-19 conditions, the patient was admitted with a possible diagnosis of super infection sinusitis and covid-19 reinfection. The samples were sent for Covid-19 -PCR testing. Due to the delay in the PCR test report (about 24–48 hours), and the severe spread of the disease in Iran, the initial management was based on Covid-19 treatment. Therefore, the patient was treated with intravenous fluid therapy, oxygen therapy, dexamethasone (8 mg daily), Remdesevir, enoxaparin (40 mg daily, SC injection), and Insulin.

**Table 1:** Initial laboratory tests.

Lab Parameter	Result
WBC	27400 u/microliter
HCT	37.8%
PLT	301000 u/microliter
HgB	12.3 g/dl
Lymphocytes	4.3%
Neutrophils	89.2%
Blood sugar	292 mg/dl
Urea	119 mg/dl
Creatinine	2.6 mg/dl
LDH	682 IU/L
K	4.3 mmol/L
Na	127 mEq/L
C.R.P	Negative
CPK	40 IU/L
Troponin	Negative
pH	7.35
pCO_2_	40.1 mmHg
Base excess	−3.9
HCO_3_	21.7 mEq/L
PTT	34 sec
INR	1.32
Urine analysis report	
Albumin (urine)	+
Bacteria	Many
WBC (urine)	8–10
Urine culture	E.Coli

**Abbreviations:** White blood cells (WBC), Hematocrit (HCT), Platelet count (PLT), Hemoglobin (Hgb), Lactic acid dehydrogenase (LDH), Potassium (K), Sodium (Na), C-Reactive protein (CRP), Creatine phosphokinase (CPK), power of hydrogen (pH), partial thromboplastin time (PTT), international normalized ratio (INR).

On the third day of admission, the PCR test reported negative result, so Covid-19 was rule out and medications that prescribed for Covid-19 (Remdesevir, dexamethasone, etc) were discontinued. The patient presented headache and pain in her upper jaw. Physical examination showed fever, erythema, and tenderness in the right cheek.

**Fig. 1:**
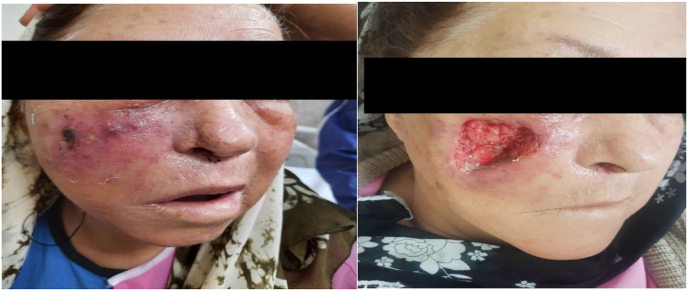
The lesion appearance.

**Fig. 2:**
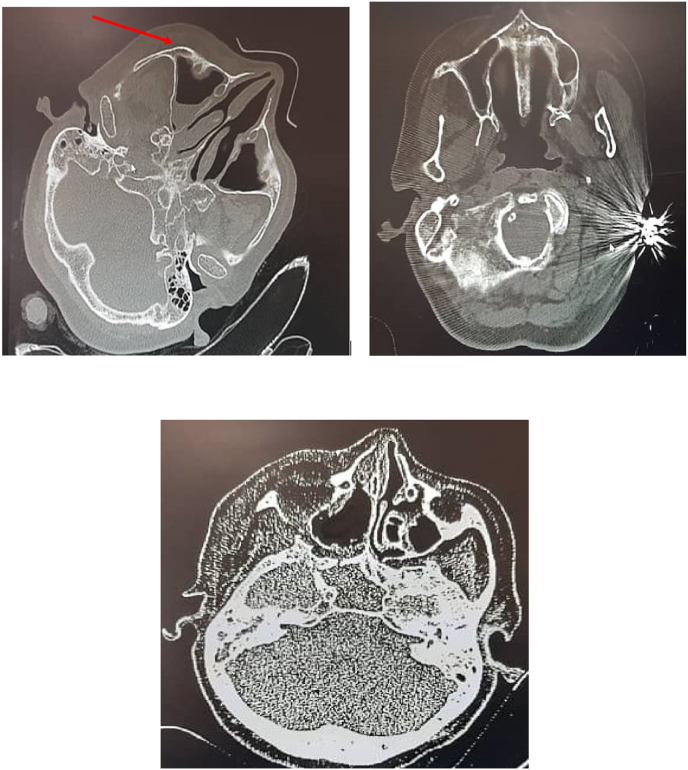
CT-scan of the brain, orbits, and paranasal sinuses.

**Fig. 3:**
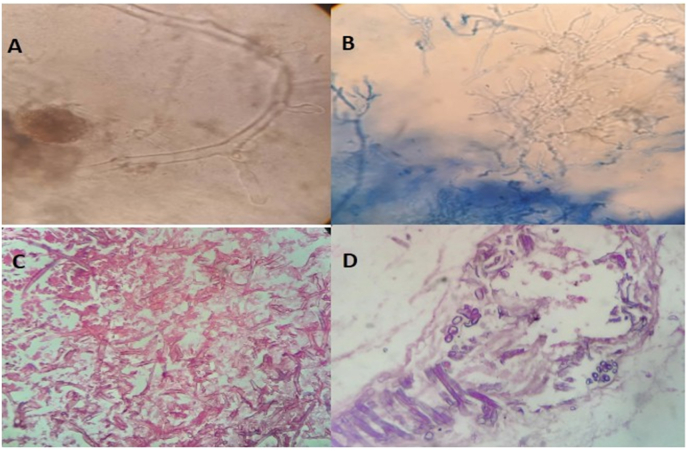
The Direct examination and pathological results confirming mucormycosis.

Emergency biopsy and culture from sinus by subsection to fungal infection conducted (Fig. 1). On CT-scan of the brain, orbits, and paranasal sinuses reveal soft tissue swelling and development of fungal invasion to the right side of the face (Fig. 2). The diagnosis of fungal infection confirmed by the positive result of biopsy direct KOH examination of sinus tissue and positive culture. Consult with ophthalmologist and otolaryngologist conducted result demonstrated intact optic nerve. Direct potassium hydroxide (KOH) examination showed aseptate branched hyphae. Furthermore, the lactophenol cotton blue (LPCB) wet mount preparation of culture has shown broad aseptate hyphae. Finaly mucormycosis confirmed by histopathologic examination using hematoxylin and eosin (H&E) staining. Although the Periodic acid–Schiff (PAS) staining was also confirming mucor hyphae (Fig. 3). Treatment with Amphotericin B as the main medication for fungal infection and ceftriaxone, clindamycin, and vancomycin as bacterial prophylaxis was started. After five days' fever was disappeared, brain MRI preformed for evaluation orbit involvement but the involvement was limited to ethmoid sinus, sometimes debridement was conducted for the patient. However, after some days’ treatment with Amphotericin B, blood urea nitrogen, and creatinine raised and the patient developed diarrhea and became hemodynamically unstable. In the stool analysis, Strongyloides stercoralis was reported. Unfortunately, despite all the treatment, the patient died on day thirty-two of this admission.

Direct potassium hydroxide (KOH) examination showing aseptated branched hyphae(A), the lactophenol cotton blue (LPCB) wet mount preparation of culture showing branched aseptate hyphae (B), histopathologic examination using hematoxylin and eosin (H&E) staining (C), the Periodic acid–Schiff (PAS) staining (D).

## 3 Discussion

We reported the patient with mucormycosis after recovery from Covid-19 infection. In initial examinations and clinical history, her symptoms were shortness of breath, dyspnea, nausea, vomiting, myalgia weakness and lethargy without chest pain and palpitation. Also, a wound with redness on the right cheek was observed. Due to the Covid-19 global pandemic and her symptom, we initially suspected the Covid-19 infection, but after PCR testing report and other laboratory findings, this diagnosis was rule out. After three days, the patient presented fever, erythema, and tenderness in the right cheek, headache and pain in her upper jaw.

Most symptoms of mucormycosis include, fever, one-sided facial swelling, excessive redness, or swelling around a wound, headache, cough, chest pain, shortness of breath, nausea and vomiting. In the recent study, the early manifestation of mucormycosis in 73% of patients was orbital apex syndrome [14].

For our patient, we used culture, CT scan and specific fungal tests. The diagnosis of mucormycosis based on detecton of organisms in tissue by histopathology or with culture confirmation. Rhino-orbital-cerebral infection, pulmonary and gastrointestinal infection are the most common mucormycosis infection. Endoscopic evaluation of the sinuses and computed tomography (CT) scan should be performed to look for tissue necrosis and eye or brain development [15].

Recent reports indicated the association between Covid-19 and mucormycosis. Several cases of mucormycosis in people with Covid-19 have been increasingly reported world-wide, in particular from India [16]. However, the frequency of Covid-19 -associated aspergillosis and candidiasis as the most frequent fungal complications in hospitalized Covid-19 patients has been highlighted. In some reports, the mean interval time between Covid-19 and mucormycosis was 1–37 days [17]. Mucormycosis is a dangerous and life-threatening disease, and its rapid diagnosis is so important. Also, fungal infection may not easily diagnose which can further delay diagnosis. On the other hand, Covid-19 may associated with many nonspecific sign and symptoms. These finding may overlap with other infections [18]. For confirming the diagnosis of Covid-19, the complete clinical and laboratory conditions (such as PCR) must be considered.

Many risk factors may involve in Covid-19 and mucormycosis. Corticosteroid therapy and diabetes mellitus augmenting the risk of mucormycosis in a susceptible individual [19]. Our patient was poor diabetic control and used corticosteroid for long time.

The primary reason that appears to be facilitating Mucorales spores to germinate in people with Covid-19 is an ideal environment of low oxygen (hypoxia), high glucose (diabetes, new-onset hyperglycemia, steroid-induced hyperglycemia), acidic medium (metabolic acidosis, diabetic ketoacidosis [DKA]), high iron levels (increased ferritins) and decreased phagocytic activity of white blood cells (WBC) due to immunosuppression (Covid-19 mediated, steroid-mediated or background comorbidities) coupled with several other shared risk factors including prolonged hospitalization with or without mechanical ventilators [20]. In the other hand, immunosuppressed individuals, are at risk for developing Strongyloides infection [21].

For future prospects, in patients with prolonged mucormycosis infection, the development of opportunistic infections such as strongyloidiasis should not be neglected. The authors suggest that, a single dose of ivermectin as strongyloidiasis prophylaxis should be given if the duration of the illness is prolonged. On the other hand, in patients with rhino-orbital mucormycosis infection, pulmonary infection also must be investigated. The CT scan can be useful in this regard.

There were limitations to our patient evaluation and management. First, delay in PCR test response and overlap of early symptoms, led to late detection of fungal infection. Second, no antifungal susceptibility testing or serum tests, such as the 1,3-Beta-D-glucan assay and the aspergillus galactomannan assay tests measurements, were performed. Third, we did not used PCR test or other molecular diagnostic to verify the fungal infection. Eventually, the fact that, it is a case report and thus the sample size is small.

## 4 Conclusion

In conclusion, this report highlighted that the medical staff, must avoid overreacting to Covid-19, and besides carefully evaluating the patient's condition. Also, clinicians must be more alert about mucormycosis especially during the first month after Covid-19 in diabetic and immunocompromised patients. Poor control of diabetes mellitus seems to be important predisposing factor.

## Conﬂict of interest

The authors confirm that this article content has no conflict of interest.

## Funding sources

This research received no funding.

## Ethics approval

The study was approved by our local ethics committee.

## Informed consent

Written informed consent was obtained from the patient for publication of this case report and accompanying images. A copy of the written consent is available for review by the Editor-in-Chief of this journal on request.

## Authorships

Sh. A, F.A involved in interpretation and collecting of data, and editing the manuscript. S.J.B, S.K and A.P involved in writing, editing and preparing the final version of manuscript. All authors reviewed the paper and approved the final version of the manuscript**.**

## Registration of research studies

1. Name of the registry:
2. Unique Identifying number or registration ID:
3. Hyperlink to your specific registration (must be publicly accessible and will be checked):

## Data availability statement

The data are available with the correspondence author and can be achieved on request.

## Guarantor

Dr Azalia Pakzad.

## Provenance and peer review

Not commissioned, externally peer-reviewed.
